# Exosomes are natural carriers of exogenous siRNA to human cells *in vitro*

**DOI:** 10.1186/1478-811X-11-88

**Published:** 2013-11-18

**Authors:** Tatyana A Shtam, Roman A Kovalev, Elena Yu Varfolomeeva, Evgeny M Makarov, Yury V Kil, Michael V Filatov

**Affiliations:** 1 Division of Molecular and Radiation Biophysics, SFBI Petersburg Nuclear Physics Institute, Gatchina 188300, Russia; 2 School of Health Sciences and Social Care, Brunel University, Uxbridge UB8 3PH, UK; 3 Department of Biophysics, St. Petersburg State Polytechnical University, St. Petersburg 195251, Russia

**Keywords:** Exosomes, RNA interference (RNAi), Drug delivery system, Cancer therapy, RAD51

## Abstract

**Background:**

Exosomes are nano-sized vesicles of endocytic origin that are involved in cell-to-cell communication including shuttle RNA, mainly mRNA and microRNA. As exosomes naturally carry RNA between cells, these particles might be useful in gene cancer therapy to deliver therapeutic short interfering RNA (siRNA) to the target cells. Despite the promise of RNA interference (RNAi) for use in therapy, several technical obstacles must be overcome. Exogenous siRNA is prone to degradation, has a limited ability to cross cell membranes and may induce an immune response. Naturally occurring RNA carriers, such as exosomes, might provide an untapped source of effective delivery strategies.

**Results:**

This study demonstrates that exosomes can deliver siRNA to recipient cells *in vitro*. The different strategies were used to introduce siRNAs into human exosomes of various origins. The delivery of fluorescently labeled siRNA via exosomes to cells was confirmed using confocal microscopy and flow cytometry. Two different siRNAs against *RAD51* and *RAD52* were used to transfect into the exosomes for therapeutic delivery into target cells. The exosome-delivered siRNAs were effective at causing post-transcriptional gene silencing in recipient cells. Moreover, the exosome-delivered siRNA against *RAD51* was functional and caused the massive reproductive cell death of recipient cancer cells.

**Conclusions:**

The results strongly suggest that exosomes effectively delivered the siRNA into the target cells. The therapeutic potential of exosome-mediated siRNA delivery was demonstrated *in vitro* by the strong knockdown of *RAD51*, a prospective therapeutic target for cancer cells. The results give an additional evidence of the ability to use human exosomes as vectors in cancer therapy, including RNAi-based gene therapy.

## Background

The use of small interfering RNAs to induce gene silencing has opened a new avenue in drug discovery. In the past decade, efforts to develop RNA-based therapeutic technologies have been significantly intensified [1-3]. Triggering RNA interference (RNAi), in particular, has become one of the most widely used techniques for biomedical applications [4-6]. RNAi uses a mechanism of posttranscriptional sequence specific gene silencing by processing double-stranded RNAs into small-interfering RNAs (siRNAs) used as part of the RNA-induced silencing complex to selectively cleave target mRNA [7]. After the discovery that synthetic siRNAs can be exogenously introduced into cells to activate RNAi [8], this approach has become a powerful method for selective suppression of specific genes of interest in different species, showing potential for use in cancer therapeutics [2,3]. However, the biomedical utility of the synthetic siRNAs is limited by several RNA structure-related factors such as the negative charge (uptake by cells that also have a negatively charged surface) and instability in the blood circulation (non-modified siRNAs have a very short half-life in the blood stream, mostly because of degradation by nucleases) [9]. Another major barrier is immunogenicity of the synthetic siRNAs or delivery vehicle, especially if repeated dosing is needed to treat disease [9,10]. These impediments can be overcome by using natural carriers of exogenous siRNA to human cells. Naturally occurring RNA carriers, such as exosomes, might provide an untapped source of effective delivery strategies [11].

Exosomes are nano-sized vesicles (30–120 nm in size) produced by many cell types, including dendritic cells (DC), B cells, T cells, mast cells, epithelial cells and tumor cells [12-15]. These vesicles are formed by inward budding of late endosomes and are then released to the extracellular environment upon fusion with the plasma membrane [15]. They have been detected in body fluids such as peripheral blood, urine, malignant effusions and bronchoalveolar lavage fluid [12,14,15]. It has been shown that these vesicles are involved in signal transduction, antigen presentation to T cells and tolerance development [12,15]. In 2007, it was demonstrated that exosomes derived from various cell types contain a substantial amount of RNA (mainly mRNA and microRNA) [16]. More importantly, the exosome vesicles shuttle RNA between cells, an activity which served as a novel means of cell-to-cell communication [12,17]. The RNA present in exosomes was therefore termed exosomal shuttle RNA (esRNA) [17]. As exosomes naturally carry RNA between cells, it has been speculated that this property might be useful in gene therapy, in which a vector is used to deliver therapeutic nucleic acids to the patient’s target cells [18-20]. In this report, we examined whether human exosomes can deliver exogenous nucleic acids to recipient cells in vitro.

In 2008, we investigated survivability of human cells and their ability to pass through the cell cycle after suppressing the homologous recombination genes by gene-specific siRNAs [21]. We demonstrated that in most cancer cell types studied the decrease in the RAD51 protein level induced cell accumulation in S and G2 phases of the cell cycle and ultimately led to the massive reproductive cell death. Our data pointed to RAD51 as a potential target for repressing the growth of abnormally proliferating cells [21]. Here, we show that the exosome-delivered siRNA against RAD51 was also functional and caused the massive reproductive cell death of recipient cells. These findings suggest that exosomes derived from human cells can be utilized in gene cancer therapy to provide target cells with heterologous nucleic acids such as therapeutic siRNAs.

## Results

### Introduction of siRNA into exosomes and delivery of heterologous siRNAs to recipient cells via exosomes

**Figure 1:**
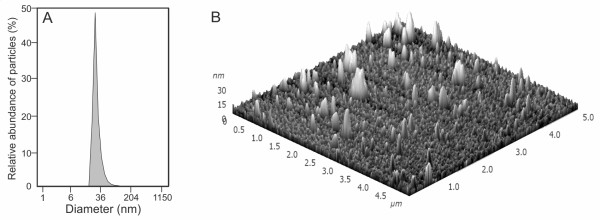
**Characterization of exosomes originating from malighnant ascitic fluid. A**, the sizes of all particles in the ultracentrifuge pellet were determined using a Microtrac S3500 particle analyzer. **B**, exosomes observed under atomic force microscopy (AFM), area scan is 5×5 micrometers.

Exosomes originating from HeLa and ascites were isolated by ultracentrifugation, as described earlier [22]. Size determination of isolated exosomes was performed using a Microtrac S3500 particle analyzer (Microtrac Inc) according to the manufacturer’s instructions (Figure 1A). The ultracentrifuge pellets of exosomes contained spherical particles that were in accordance with the exosome size range described earlier [12,15,22]. To estimate the homogeneity of the particles the atomic force microscopy method was used. The exosomes appeared as homogeneous circular bulging vesicular structures (Figure 1B). We also confirmed the presence of exosomal marker proteins HLA-ABC and CD63 [15] on the surface of these vesicles by dot blot analysis.

**Figure 2:**
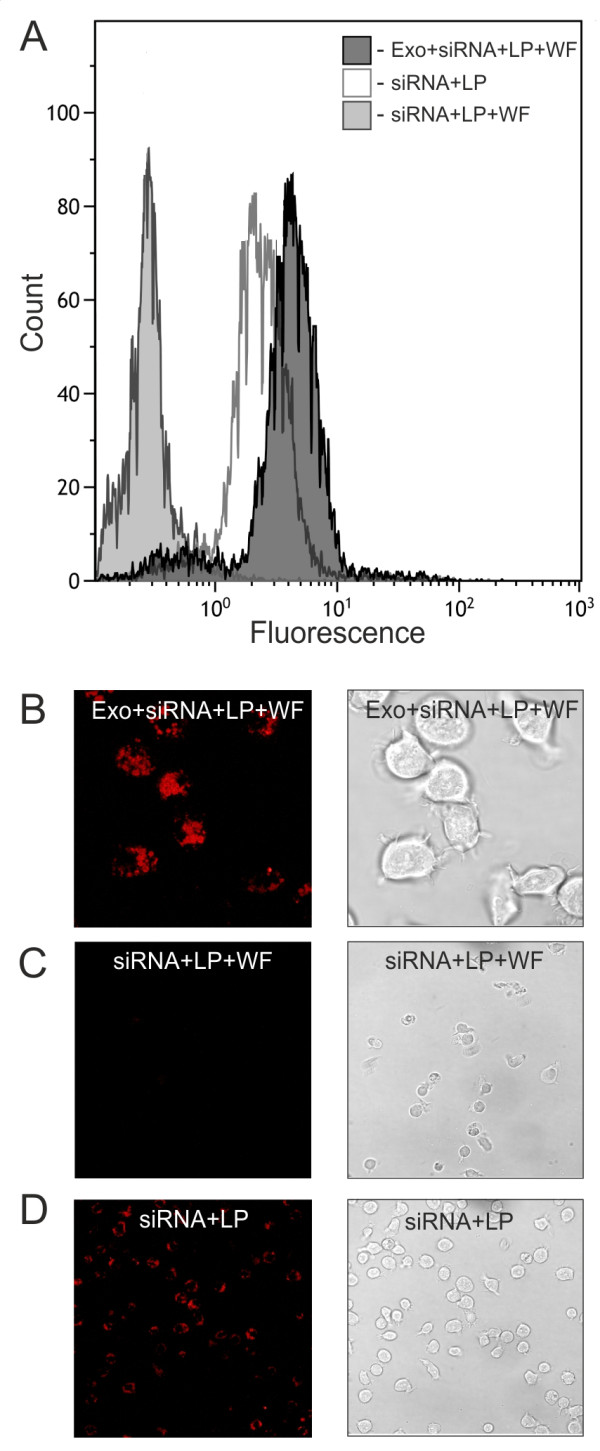
***In vitro *delivery of fluorescently labeled siRNA via chemically loaded exosomes.** The recipient cells were treated for 24 h with exosomes loaded with Lipofectamine-formulated siRNA and washed several times through a 100-kDa filter (Exo + siRNA + LP + WF). The sample prepared in identical way but without adding of exosomes was used (siRNA + LP + WF) as a control. The cells were also transfected with siRNA by standard procedure (siRNA + LP). **A**, flow cytometry analysis of HeLa cells transfected by siRNA. **B-D**, representative images of HeLa cells 24 h after transfection.

Next, we investigated the possibility of loading exosomes with exogenous cargoes using chemical treatment. As chemical transfection for membrane particles at the nanometer scale is not well-characterized, nonspecific Alexa flour 488 labeled siRNA (siRNA AF488) was used for the empirical optimization of the protocol. To examine the efficiency of chemical loading of exosomes, siRNA AF488 was mixed with Lipofectamine to allow for the formation of complexes (siRNA embedded in lipid micelles), which was then added to the exosomes and incubated for 30 min at RT. The mixture was purified for 3-5 times by washing and ultra-filtration through a 100-kDa filter to eliminate the excess of free siRNA as well as siRNA embedded into the micelles (Exo + siRNA + LP + WF). Exosomes loaded with siRNA were co-cultured with the recipient cells for 24 h in order to transfect cells with the heterologous siRNA. High delivery efficiency of siRNA by exosomes to recipient cells was confirmed by confocal microscopy and flow cytometry (Figure 2A,B). The recipient cells were also transfected with siRNA AF488 treated with Lipofectamine (siRNA + LP) in usual manner and after purifying for several times through a 100-kDa filter as exosomal samples (siRNA + LP + WF). The results from flow cytometry and confocal microscopy showed that the fluorescence signal of the siRNA could be detected only in the cells transfected usually (siRNA + LP), as expected (Figure 2A,D). But no fluorescence signal was detected in control cells incubated with washed and ultra-filtrated transfection complexes (siRNA + LP + WF) (Figure 2A,C), confirming that the purification procedure used was sufficient to remove all non-associated with exosomes siRNA from the samples. Thus, the results indicate the possibility of siRNA delivery into cells by exosomes in the presence of Lipofectamine. But, as siRNA embedded into the micelles could unspecifically be attached to the exosomes, it was indistinguishable whether the exosomes or the micelles of chemical transfection finally delivered exogenous nucleic acids to cells.

**Figure 3:**
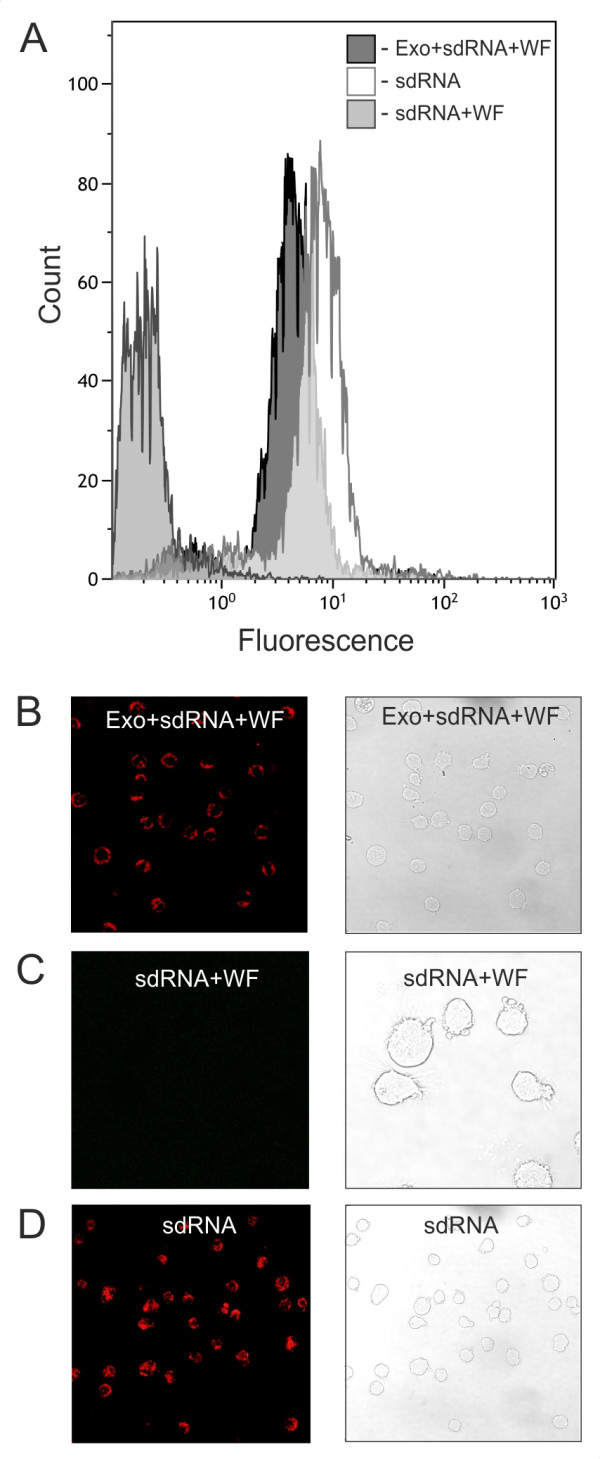
**Transfection of HeLa cells by fluorescently labeled self-delivering sdRNA via exosomes.** The recipient cells were treated for 24 h with exosomes loaded with the sdRNA and washed several times through a 100-kDa filter (Exo + sdRNA + WF). The sample prepared in identical way but without adding of exosomes was used (sdRNA + WF) as a control. The cells were also transfected with sdRNA by standard procedure (sdRNA). **A**, flow cytometry analysis of HeLa cells transfected by sdRNA. **B-D**, representative images of HeLa cells 24 h after transfection.

To examine the possibility to input siRNA into exosomes in principle we used the fluorescently labeled self-delivering sdRNA. Self-deliverable RNA molecules do not require any transfection reagent, vehicle or special cell treatment. The sdRNA was mixed with exosomes, incubated 30 min at RT and then the exosome vesicles were purified using 3-5 times washing and ultrafiltration through a 100-kDa filter (Amicon ultra, Millipore) to eliminate the excess of free sdRNA (Exo + sdRNA + WF). As a control the sample prepared in identical way without exosomes was used (sdRNA + WF). Then both samples were co-cultured with the recipient cells for 24 h. The cells were also transfected with sdRNA in usual manner (sdRNA). The results from flow cytometry and confocal microscopy showed that the fluorescent signal of sdRNA was indeed associated with presence of exosomes (Figure 3A-D). SdRNA could not be detected in the cells co-cultured with control sample without exosomes, confirming that the excess of free sdRNA was depleted by purifying (Figure 3C). In summary, the results strongly suggest that the approach used was successful at introducing the heterologous siRNAs into the exosomes and at delivery of the siRNAs to recipient cells via exosomes.

### Exosome-delivered siRNA is functional in recipient cells

Given that exosomes from HeLa and ascites could act as a carrier to deliver nonspecific heterologous genetic material to recipient cells, we investigated whether the specific siRNAs delivered via exosomes to cells would down-regulate the expression of target genes and, if so, whether the exosome-delivered siRNA would be functional in recipient cells. Therefore, we targeted *RAD51* and *RAD52* genes by specific siRNAs delivered via exosomes to HeLa and HT1080 cells to evaluate the therapeutic potential of this technology. The RAD51 recombinase executes the central functions in homologous recombination: the search for a homologous template DNA and the formation of the joint heteroduplex molecule between the damaged DNA and the undamaged template [23]. In addition to RAD51, homologous recombination requires the coordinated action of a number of other proteins of homologous recombination, including RAD52, which can bind DNA ends and anneal complementary single-stranded DNA molecules [24]. The role of homologous recombination in the maintenance of stable genome and viability of somatic mammalian cells is still under investigation. We have shown previously that depression of the *RAD51* gene function leads to the massive reproductive death of human cancer cells in the absence of genotoxic injuries [21]. Our data demonstrated that the significant down-regulation of RAD51 but not RAD52 protein by the specific siRNA resulted in S/G2 cell cycle blocks. In most of the cancer cell lines such blocks resulted in dramatic decrease in cell viability accompanied by apoptosis or irreversible loss of their ability to proliferate. Therefore, we pointed to RAD51 as a potential target to depress the abnormally proliferating cells [21]. Here *RAD51* and *RAD52* were knockdown by specific siRNAs in HeLa and HT1080 cells via genotoxic delivery by exosomes derived from HeLa and ascitic fluids. Both cell lines were co-cultured with exosomes chemically loaded with the RAD51 or RAD52 siRNA for 72-96 h. Western blot analysis showed a considerable reduction in both RAD51 and RAD52 protein levels in cells as after transfection with specific siRNAs via exosomes and after siRNA standard cell transfection with Lipofectamine (Additional file 1: Figure S1A). Moreover the recruitment of RAD51 at double-strand breaks induced in HeLa cells by ionizing radiation was reduced in cells treated by exosomes loaded with anti-RAD51 siRNA (Additional file 1: Figure S1B).

**Figure 4:**
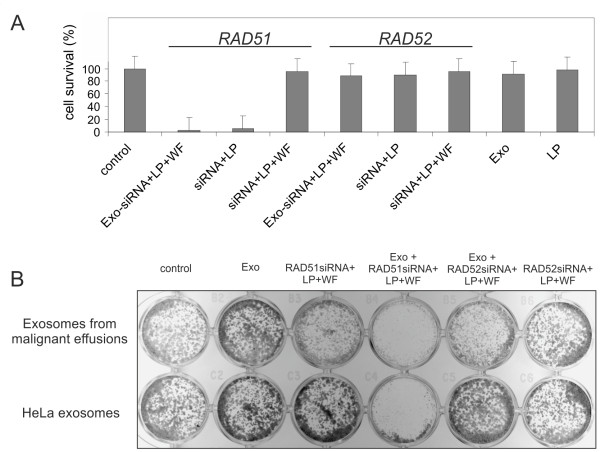
***RAD51 *down-regulation by exosome delivered siRNA leads to dramatic decrease of viability of human cells. A**, the survival of HeLa cells after RAD51 and RAD52 depletion by two ways: by direct specific siRNA chemical transfection (siRNA + LP) and by chemically loaded with siRNA exosomes (Exo + siRNA + LP + WF). The survival of the non-transfected cells was assumed as 100% (control). Error bars represent the standard error of the mean (SE) of at least three independent experiments. **B**, the example of clonogenic cell survival of the HeLa cells after co-culturing with ascites-derived exosomes (upper row) or HeLa exosomes (bottom row) which act as a carrier to deliver specific siRNAs to recipient cells.

Next we examined the colony forming ability of *RAD51* or *RAD52* knocked down cells. The total amount of colonies was counted in 5-7 days after siRNA was added (Figure 4). Standard transfection with siRNA-antiRAD51 resulted in a significant decrease of HeLa and HT1080 cell survival (siRNA + LP). At the same time transfection with siRNA-antiRAD52 had no effect on the cell survival. The same results were observed for the cells transfected by the siRNAs via exosomes (Exo + siRNA + LP + WF). Neither Lipofectamine alone (LP) nor exosomes alone (Exo) nor purified by washing and filtering siRNAs samples (siRNA + LP + WF) had significant effect on the cell survival.

**Figure 5:**
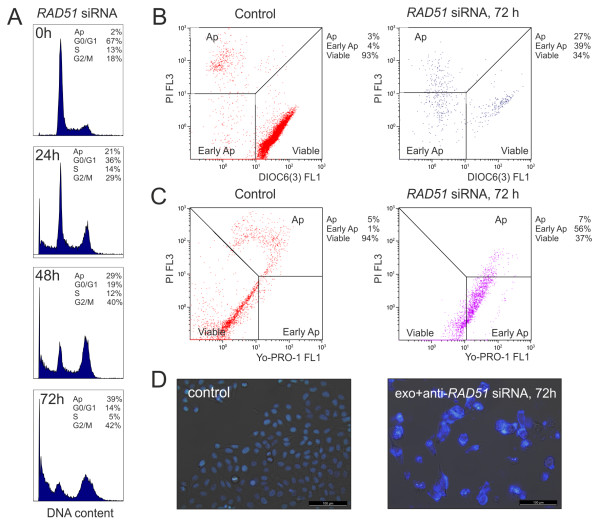
**Exosome-delivered RAD51 siRNA induced accumulation of cells in G2/M phase of cell cycle and resulted in apoptosis of the recipient cells. A**, Cell cycle distribution of HeLa cells treated by RAD51 siRNA via exosomes the indicated time. Examination of Hoechst 33342 staining revealed typical cell cycle profiles. It was possible to identify G0/G1 peak, G2/M peak, S phase and dead/apoptotic cells (Ap) as a sub-G1 population. The fraction of cells in each cell cycle phase is indicated at the top. **B** and **C**, cytometric analyses of mitochondrial transmembrane potential and plasma membrane integrity fluorochromes after RAD51 depletion. HeLa cells were incubated for 72 h with or without (control) exosomes loaded by RAD51 siRNA. DiOC6(3)/PI double staining flow cytometry analyses **(B)**. Yo-Pro-1/PI double staining flow cytometry dot plots **(C)**. **D**, the cells incubated with exosomes loaded by RAD51 siRNA were confirmed to be dead or apoptotic by microscopy observation of nuclear fragmentation with the Hoechst 33342 staining.

We also examined the influence of the RAD51 protein on the cell cycle parameters. As shown in Figure 5A, silencing of the *RAD51* gene via the siRNA loaded exosomes during 24-48 h induced the accumulation of S-phase and G2-phase cells. More prolonged RAD51 siRNA transfection via exosomes (72 h) caused the block of the recipient cells mainly in the G2/M phase and apoptotic cells death, which was indicated by high degree of cell DNA degradation (Figure 5A). Apoptosis was also studied by flow cytometry with mitochondrial membrane potential and membrane integrity fluorochromes. Both DiOC6(3)/PI and Yo-Pro-1/PI double staining flow cytometry showed that the apoptotic rate of HeLa cells transfected by RAD51 siRNA via exosomes was increased compared with that in the control cells (Figure 5B,C). In addition, cell morphology was determined in RAD51 depleted cells. Figure 5D shows the morphology of HeLa cells stained with Hoechst 33342. The confocal images showed that control cells possessed intact nuclei. The RAD51 siRNA transfection via exosomes caused the formation of degraded nuclei, membrane blebbing and clear apoptotic bodies (Figure 5D).

Thus, exosomes from HeLa and ascites were able to deliver heterologous siRNAs to human cells. Importantly, the exosome-delivered RAD51 siRNA was functional and caused post-transcriptional gene silencing, induced accumulation of the cells in S and G2/M phases of cell cycle and resulted in recipient cell death. Interestingly, the exosomes from different ascites were able to deliver heterologous siRNAs to human cultured cells such as HeLa or HT1080 cells (Figure 4B). This observation indicates the nonspecific ability of the cells to capture alien vesicles.

**Figure 6:**
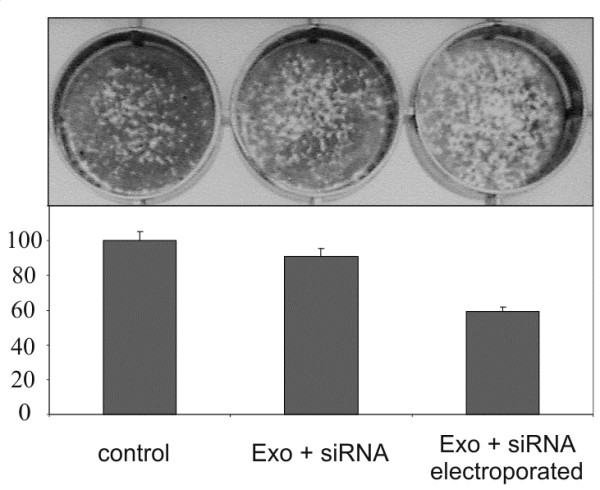
**Delivery of anti-RAD51 siRNA to HeLa cells via exosomes.** HeLa cells were grown with exosomes loaded with RAD51 siRNA by electroporation (Exo + siRNA electroporated). Non-targeted cells were used as negative control (control) in addition to cells grown with non-electroporated mixture of anti-RAD51 siRNA and exosomes (Exo + siRNA). The introduction of siRNA into exosomes was evaluated using a survival analysis of HeLa cells after 5-7 days of co-culturing with RAD51 siRNA electroporated exosomes as compared to non-electroporated mixture of siRNA and exosomes. The survival of the non-transfected cells was assumed as 100%. Error bars represent the standard error of the mean (SE) of at least three independent experiments.

As mentioned above, we could not exclude adhesion of siRNA embedded into the micelles on the surface of exosomes in the presence of Lipofectamine. However, the location of siRNA outside of exosomes would lead to problems in delivery of safe siRNA *in vivo*. Therefore, electroporation was examined as a means of introducing genetic material into exosomes. Having discovered the optimal parameters for transferring siRNA into exosomes by electroporation, siRNA against *RAD51* was packaged into exosomes derived from HeLa cells. Survival of the cells after co-culturing with electroporated exosomes was used as a test system to optimize the transfection efficiency*.* The results shown in Figure 6 provide evidence that the heterologous siRNA was introduced into exosomes by electroporation. Summing up the data, we can assume that exosomes effectively delivered the siRNA into the target cells, causing selective genes silencing and leading to reproductive cancer cell death by knockdown of *RAD51*.

## Discussion

The identification of extracellular phospholipid vesicles as conveyors of cellular information has created excitement in the field of drug delivery [12]. Biological therapeutics, including short interfering RNA are prone to degradation, have limited ability to cross biological membranes, and may elicit immune responses [3]. Therefore, delivery systems for such drugs are under intensive investigation [25]. Exploiting extracellular vesicles as carriers for biological therapeutics is a promising strategy to overcome these issues and to achieve efficient delivery to the cytosol of target cells [19]. Exosomes are a well-studied class of extracellular vesicles known to carry proteins and nucleic acids, making them especially suitable for such strategies. Exosomes and microvesicles are naturally adapted for the transport and intracellular delivery of proteins and nucleic acids. This makes them particularly attractive for the delivery of pharmaceutical proteins and nucleic acids, such as siRNA [19,25]. Intracellular delivery of siRNA is a challenging task, given that naked siRNAs are rapidly degraded in the circulation, their size and negative charge limits membrane passage and cellular uptake, some siRNA sequence motifs may elicit undesired immune responses, and targeting to specific tissues and cells is required to reduce adverse effects caused by off-target silencing [26,27]. Encapsulation of nucleic acid-based therapeutics in endogenous transporting vesicles is a promising novel strategy to overcome most of these delivery issues. Exosomes may be most suitable for such strategies, because they are small (30-120 nm), relatively homogenous in size, and well studied. Their size is advantageous for their use as drug delivery systems, because this allows them to evade rapid clearance by the mononuclear phagocyte system and enhances passage through fenestrations in the vessel wall, as might occur during inflammation [28].

The successful use of exosomes for the targeted delivery of siRNA has been recently demonstrated by Alvarez-Erviti et al. [29]. They harvested dendritic cells from mice and transfected them to express the neuronal targeting ligand, RVG, coupled to the exosomal membrane protein, Lamp2b. This protein was expressed by the cells and incorporated in secreted exosomes. The exosomes were harvested, purified, and loaded with siRNA against an important protein in Alzheimer pathogenesis (BACE1) by electroporation. When the modified exosomes were injected intravenously in wild-type mice, a 60% decrease of BACE1 mRNA in the brain cortex was observed after 3 days. Moreover, no increase in serum interleukin-6, interferon gamma-induced protein 10, tumor necrosis factor alpha and interferon alpha concentrations was observed after injection of the exosomes, suggesting that the modified exosomes were immunologically inert. The biotechnological approach to create exosome-based delivery systems used by Alvarez-Erviti et al. was the first demonstration of an exosome-based drug delivery system which showed efficient in vivo delivery of siRNA [29]. In 2012, Wahlgren et al described an “exosome display technology in vitro” in which, exogenous siRNAs were successfully introduced into various kinds of human exosomes and were used to deliver siRNA to human mononuclear blood cells. Plasma exosomes effectively delivered the siRNA into the target cells, causing selective gene silencing of MAPK-1 [30]. Other strategies to exploit exosomes for siRNA delivery *in vitro* and *in vivo* have also been reported [25,31,32]. But, despite promising uses for exosomes as a delivery of exogenous RNA, to date, only a few research reports on the subject were published.

The purpose of this study was to investigate the potential use of exosomes as a carrier for siRNA delivery *in vitro*. Fluorescently labeled siRNAs were successfully introduced into various kinds of human exosomes: conventional siRNA was loaded with a chemical transfection reagent (Lipofectamine) and self-delivering sdRNA without any reagent. Both cultural and ascites-derived exosomes effectively delivered the hydrophobically modified sdRNA or usual siRNA molecules into the different target cells. This observation indicates the nonspecific ability of the cells to capture alien vesicles. Taken together, the results strongly suggest that the approaches used were successful at introducing the heterologous siRNAs into the exosomes and at delivery of heterologous siRNAs to recipient cells via exosomes.

Next, we examined if exosome-delivered siRNA was functional and caused post-transcriptional gene silencing in recipient cells. The *RAD51* gene was found to be down-regulated in HeLa and HT1080 cells that had been co-cultured with exosomes containing siRNA against the *RAD51* transcript. The results show that exosomes effectively delivered the siRNA against RAD51 into the target cells, causing selective genes silencing and leading to reproductive cancer cell death by knockdown of RAD51. The effect of *RAD51* gene knockdown was equally visible in HeLa and in HT1080 cells. This indicates approximately the same ability of these cancer cell types to adsorb exosomes. Our results, and the results published previously [30], show that chemical loading of the exosomes to be used as siRNA delivery vector was inapplicable as the excess of micelles (siRNA embedded in lipid micelles) could not be separated from the exosomes. It is uncertain whether the exosomes or the excess of micelles deliver the nucleic acid of interest to cells and, therefore, safe delivery of siRNA via exosomes is not evident in the long term use this approach *in vivo*. Thus, electroporation was examined as a means of introducing siRNA against RAD51 into exosomes. Survival of HeLa cells after co-culturing with electroporated exosomes was used as a test system to optimize the transfection. The results provide sufficient evidence that the heterologous siRNA was quite effectively introduced into exosomes using electroporation, but the method may need to be optimized for each exosome and each cell type.

## Conclusions

We can assume that exosomes effectively deliver siRNA into the target cells *in vitro*, causing selective genes silencing and leading to reproductive cancer cell death by knockdown of RAD51 recombinase. Taken together, (i) exosomes may represent an efficient delivery platform for siRNAs, (ii) siRNA-mediated induction of RNAi is a promising approach for the knockdown of pathologically relevant oncogenes, and (iii) delivery of siRNA via exosomes may become an attractive therapeutic strategy for the treatment of cancer.

## Methods

### Exosome purification

For the generation of exosomes HeLa and HT1080 human fibosarcoma cells were grown during 5 days in 30 ml (250 sm^2^ flacks were used) of DMEM-F12 medium supplemented with 10% fetal bovine serum (FBS) depleted from exosomes as described [22]. On the day of collection, the medium from the cells was removed (conditioned medium). Exosomes were isolated by differential centrifugations and micro-filtration as previously described [15,22]. Briefly, 100 ml of cell supernatants were harvested, centrifuged at 300 g for 10 min to eliminate cells and then at 15 000 g for 30 min to remove cell debris. Exosomes were pelleted by ultracentrifugation at 110 000 g for 70 min at 4°C. The exosome pellet was resuspended in 1 ml of DMEM medium by syringing three to five times through a sterile 27-gauge needle to prevent the exosomes from clumping together. Then the exosome samples were filtered through a 0.22-μm filter for sterilization. The protein content of the exosome suspension was quantified using the Bradford reagent. Exosomes from malighnant ascitic fluids (ascites) were prepared by differential centrifugation and filtration as for HeLa and HT1080 cells.

### Atomic force microscopy (AFM)

Purified exosomes were diluted in de-ionized water and adsorbed to freshly cleaved mica sheets, fixed with 0.5% glutaraldehyde, rinsed with de-ionized water and air-dried. The samples were scanned in the air by semi-contact method with a scanning microscope of Solver Bio series (NT-MDT, Russia) equipped with silicon test probe (type NSG01), with a characteristic stiffness of 5.5 N/m and a typical radius of curvature of the tip (less than 10 nm). The initial amplitude of scanning was set to 16 nA in current terms; SetPoint was adjusted to half of the amplitude. Scanning was performed with a frequency of 1.01 Hz. The images were processed using standard software package (Image Analysis Nova).

### Loading of exosomes with siRNA

Two different methods, chemical treatment and electroporation, were used. For chemical loading of exosomes, siRNA at a final concentration of 2 μmol/ml was mixed with Lipofectamine transfection reagent in 100 μl of siRNA buffer and incubated for 10 min at RT. Then 300 μl of exosome suspension was added and the mixture was incubated for additional 30 min at RT. To remove the excess of micelles, the exosomes were purified using three to five times filtration through a 100-kDa filter (Amicon, Millipore) with washing by cultural medium.

For electroporation assays exosomes derived from HeLa, HT1080 or ascitic fluids were diluted in cytomix transfection buffer. SiRNAs against RAD51 or RAD52 at a final concentration of 2 μmol/ml were added to 300 μl of exosome sample. The mixtures were transferred into ice cold 0.4-cm cuvettes and electroporated at 0.7 kV using 350 microsecond pulse 20 times by the Eppendorf multiporator.

RNA duplexes targeting RAD51 or RAD52 (ON-TARGETplus SMARTpool) were purchased from Dharmacon. For control experiments fluorescently labeled conventional siRNA (Qiagen) and self-delivering siRNA (sdRNA) were used. SdRNA, a hydrophobically modified siRNA for cell transfection without formulation with Lipofectamine, was kindly provided by Advirna, Cambridge MA.

### Transfection of siRNA into cells via exosomes

For siRNAs exosome-delivery assays, HeLa and HT1080 cells were seeded in 24-well plates at a density of 0.5 × 10^4^ cells/well. A 30 μl of purified samples, containing exosomes loaded with siRNA were co-cultured with the recipient cells in order to deliver the heterologous siRNA. The cells, transfected with a fluorescently labeled siRNA, were trypsinized, then isolated, washed three times with PBS and were analyzed by Confocal microscopy (LEICA TCS SP5X) and Flow cytometry (Beckman Coulter). All tissue culture reagents were obtained from Invitrogen and Hyclone.

### Protein detection

Dot blot was used to detect exosomal proteins HLA-ABC and CD63 in exosome suspension after purification. Fifteen micrograms of exosome suspension were dropped into nitrocellulose (NC) membranes (Millipore). The membranes were dried, blocked with 5% nonfat dry milk, washed in Tris-Buffered Saline with 0.1% Tween 20 buffer (TBST) and probed with primary antibodies against HLA-ABC (1:200 dilution) or CD63 (1:200 dilution). All the primary antibodies were obtained from Beckman Coulter. After washing in TBST, membranes were incubated with HRP-conjugated secondary antibody (antimouse IgG: 1:10000; Sigma). For detection, enhanced chemiluminesence was carried out using the ECL plus kit (Amersham Biosciences Corp).

The exosome-based delivery of siRNA to cells was determined by Western Blot analysis and compared with direct transfection of the same siRNA to cells. For expression analysis of RAD51 and RAD52 proteins whole cell extracts were made by lysis of 1-5 × 10^6^ cells in 30–50 μl of lysis buffer (10 мМ Tris-HCl pH 7.4, 0.1% Triton X-100, 5 мМ PMSF, 5 мМ MgCl_2_, 5 u/ml DNAse I, 20 мМ β-mercaptoethanol). For western analysis aliquots of extracts containing equal amounts of total protein as determined by Bradford reagent were used. Following sonication and boiling, aliquots were resolved by 12% SDS–PAGE according to Laemmli, transferred to a PVDF membrane (Thermo Scientific) and hybridized with a mouse monoclonal antibody against RAD51 at the 1:1000 dilution (clone 3C10, Invitrogen) or against RAD52 at the 1:1000 dilution (Thermo Scientific) followed by a peroxidase-labeled anti-mouse antibody (Sigma) at the 1:10000 dilution. Antibody binding was detected by enhanced chemoluminescence (Pierce). Equality of loading was confirmed by hybridizing with a monoclonal antibody against GAPDH at the 1:10000 dilution (Clone: ZG003, Zymed).

### Clonogenic survival assays

After treatment with exosomes loaded with siRNAs as stated above the cells were cultured for several days. After 5-7 days the plates were rinsed with PBS, stained with methylene blue (0.25%) and visible colonies were counted. The survival of the non-transfected cells was assumed as 100%.

### Cell cycle analysis

For monitoring of cell cycle parameters 30–40% confluent HeLa cells, seeded in a 35 mm diameter Petri dishes, were incubated with exosomes loaded with RAD51 siRNA for 24–72 hours. After incubation, cells were trypsinized, centrifuged at 200 g for 10 min, washed with PBS and then stained with a dye solution (1 μg/ml Hoechst 33342, 0.1% Triton X-100) for 10 min and subjected to flow cytometry analysis. Except for RAD51 siRNA, the control cells were subjected to the same experimental conditions.

### Apoptosis assay

DiOC6(3)/PI staining was used to detect mitochondrial membrane potential assessment and plasma membrane integrity. A total of 10^6^ cells were diluted in 100 μl of PBS. DiOC6(3) (Invitrogen) was added up to a final concentration of 20 nM. The tubes were then gently mixed and incubated for 20 min in 5% CO_2_ atmosphere at 37°C. Then the cells were washed with PBS, contained 2% of serum, and PI (Propidium Iodide) was added up to a final concentration of 1 μg/ml. Flow cytometry analysis was conducted within 10 min. The data from 10^4^ cells were collected and analyzed using CellQuest Pro Software (Becton Dickinson) to calculate the proportion of early apoptotic, late apoptotic/dead and viable healthy cells.

The Vybrant Apoptosis Assay Kit (Molecular Probes) was used to detect changes in plasma membrane permeability to Yo-Pro-1 related to apoptosis. A total of 10^6^ cells were diluted in 1 ml of PBS; 1 μl of Yo-Pro-1 (100 μmol/l) was added. The tubes were gently mixed and incubated for 20 min at RT and 6 μmol/l PI were added to each tube. Flow cytometry analysis was conducted within 10 min.

### Immunofluorescence analysis of RAD51 foci

Before irradiation HeLa cells were incubated for 48 h with exosomes loaded with RAD51siRNA. Gamma irradiation was performed with a ^137^Cs source. After irradiation at 10 Gy the cells were maintained for 6 h in the fresh medium and then analyzed by immunofluorescence for the presence of RAD51 foci. Immunofluorescence staining was performed by 4% PFA fixation, 0.2% Triton X-100 treatment, blocking in 1% BSA plus 1% normal goat serum and staining with first mouse monoclonal antibody against RAD51 at the 1:200 dilution (clone 3C10, Invitrogen) and Alexa Fluor 594 secondary antibodies (Invitrogen). Except for RAD51 siRNA, the control cells were subjected to the same experimental conditions.

### Endnotes

This work was performed with financial assistance of Russian Federal Program “Scientific and Scientific-Pedagogical Personnel of Innovative Russia”, contract 14.740.11.0754 and Fellowship from the Administration of Leningrad region to Shtam T.

## Abbreviations

RNAi: RNA interference; siRNAs: Small-interfering RNAs; LP: Lipofectamine; AFM: Atomic force microscopy; PI: Propidium iodide.

## Competing interests

The authors declare that they have no competing interests.

## Authors’ contributions

MVF and TAS conceived and designed the experiments. TAS, RAK and EYV carried out the experiments. EMM contributed into analysis and interpretation of data. TAS, YVK and MVF wrote the manuscript. All authors read and approved the final manuscript.

## Supplementary Material

Additional file 1: Figure S1 siRNA transfection via exosomes resulted in a substantial decrease of the protein expression level and in suppression of RAD51 downstream activity. **A**, Western blot probed with RAD51 and RAD52 in HeLa cells direct transfected with specific siRNAs and with exosome carriers of siRNAs against RAD51 or RAD52. The cells treated by Lipofectamine alone were analyzed as a control. Equality of loading was confirmed by hybridizing with a monoclonal antibody against GAPDH. **B**, Analysis of RAD51 recruitment in HeLa cells irradiated with γ-rays. Representative pictures of RAD51 repair foci in HeLa cells at 6 h after irradiation with 10 Gy, control (left panel) and cells transfected by RAD51 siRNA via exosomes (right panel). Click here for file
