# Supplementary material for: Exosomes are natural carriers of exogenous siRNA to human cells in vitro
Source: Cell Commun Signal. 2013 Nov 18;11:88. doi: 10.1186/1478-811X-11-88 (PMC3895799; doi:10.1186/1478-811X-11-88)
Supplement: Additional file 1: Figure S1 — siRNA transfection via exosomes resulted in a substantial decrease of the protein expression level and in suppression of RAD51 downstream activity. A, Western blot probed with RAD51 and RAD52 in HeLa cells direct transfected with specific siRNAs and with exosome carriers of siRNAs against RAD51 or RAD52. The cells treated by Lipofectamine alone were analyzed as a control. Equality of loading was confirmed by hybridizing with a monoclonal antibody against GAPDH. B, Analysis of RAD51 recruitment in HeLa cells irradiated with γ-rays. Representative pictures of RAD51 repair foci in HeLa cells at 6 h after irradiation with 10 Gy, control (left panel) and cells transfected by RAD51 siRNA via exosomes (right panel). [file 1478-811X-11-88-S1.pdf]

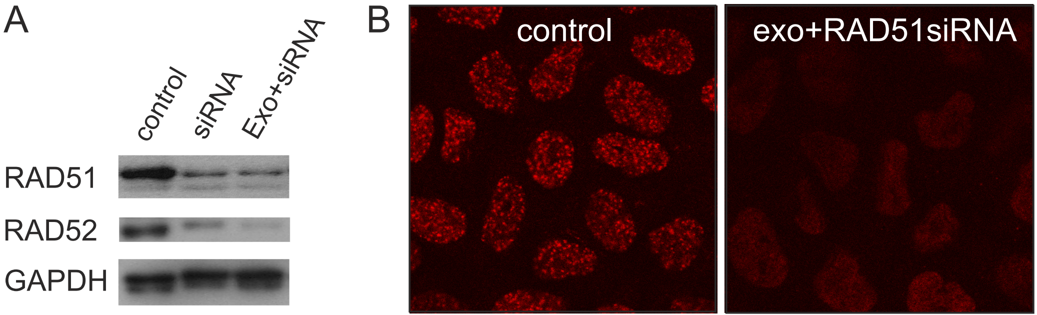

Figure S1. siRNA transfection via exosomes resulted in a substantial decrease of the protein expression level and in suppression of RAD51 downstream activity. **A**, Western blot probed with RAD51 and RAD52 in HeLa cells direct transfected with specific siRNAs and with exosome carriers of siRNAs against RAD51 or RAD52. The cells treated by Lipofectamine alone were analyzed as a control. Equality of loading was confirmed by hybridizing with a monoclonal antibody against GAPDH. **B**, Analysis of RAD51 recruitment in HeLa cells irradiated with **γ**-rays. Representative pictures of RAD51 repair foci in HeLa cells at 6h after irradiation with 10Gy, control (left panel) and cells transfected by Rad51 siRNA via exosomes (right panel).
